# Effect of quorum-quenching bacterium *Bacillus* sp. QSI-1 on protein profiles and extracellular enzymatic activities of *Aeromonas hydrophila* YJ-1

**DOI:** 10.1186/s12866-019-1515-6

**Published:** 2019-06-21

**Authors:** Shuxin Zhou, Zixun Yu, Weihua Chu

**Affiliations:** 1 Department of Pharmaceutical Microbiology, School of Life Science and Technology, China Pharmaceutical University, Nanjing, 210009 China; 2 School of Pharmacy, China Pharmaceutical University, Nanjing, 210009 China

**Keywords:** *Aeromonas hydrophila*, *Bacillus* sp., Co-culture, Proteomic, Virulence factors

## Abstract

**Background:**

In natural environments, bacteria always live in communities with others where their physiological characteristics are influenced by each other. Bacteria can communicate with one another by using autoinducers. The current knowledge on the effect of quenching bacteria on others is limited to assess the impact of quorum-quenching bacterium *Bacillus* sp. QSI-1 on proteins pattern and virulence factors production of *Aeromonas hydrophila* YJ-1*.* Proteomic analysis was performed to find out protein changes and virulence factors, after 24 h co-culture.

**Results:**

Results showed that several proteins of *A. hydrophila* YJ-1 were altered, seventy-two differentially expressed protein spots were excised from 2-DE gels and analyzed by MALDI-TOF/TOF MS, resulting in 63 individual proteins being clearly identified from 70 spots. Among these proteins, 50 were divided into 22 classes and mapped onto 18 biological pathways. Mixed-culture growth with *Bacillus* sp. QSI-1 resulted in an increase of *A. hydrophilia* proteins involved in RNA polymerase activity, biosynthesis of secondary metabolites, flagellar assembly, and two-component systems. In contrast, mixed culture resulted in a decreased level of proteins involved in thiamine metabolism; valine, leucine and isoleucine biosynthesis; pantothenate and CoA biosynthesis. In addition, the two extracellular virulence factors, proteases and hemolysin, were significantly reduced when *A. hydrophila* was co-cultured with QSI-1, while only lipase activity was observed to increase.

**Conclusions:**

The information gathered from our experiment showed that *Bacillus* sp. QSI-1 has a major impact on the expression of proteins, including virulence factors of *A. hydrophila*.

**Electronic supplementary material:**

The online version of this article (10.1186/s12866-019-1515-6) contains supplementary material, which is available to authorized users.

## Background

Quorum sensing (QS) is the regulation of gene expression in response to cell density and enables bacteria to regulate the expression of virulence factors and biofilm formation. In nature, bacteria live in mixed populations with other bacterial species. One mechanism used during bacterial species’ competition is QS inactivation, a process commonly referred to as “quorum quenching” (QQ) [1]. During QQ, the QS signal molecules can be inactivated by enzymatic degradation or modification. Such quorum quenching enzymes are wide-spread in the bacterial world and have also been found in eukaryotes. Quorum quenching enzymes can be used to combat bacterial infection [2]. The emergence of antibiotic resistant bacterial strains is a global threat to both animal and human health. The development of new and effective antibiotics is slow, thus therapies that target bacterial QS pathways without killing the bacteria are promising alternatives [3, 4].

*Aeromonas hydrophila* is an important aquatic pathogen that causes motile aeromonad septicemia in aquatic animals, resulting in great annual economic losses for the aquaculture industry worldwide [5]. Additionally, *A. hydrophila* is an important zoonotic pathogen that can cause foodborne gastrointestinal and extra-intestinal infections in humans [6]. The pathogenicity of *A. hydrophila* is closely related to the production of virulence factors and biofilm formation, with virulence factors including serine protease, hemolysin, aerolysin, enterotoxin (cytotoxic enterotoxin) and adhesins, such as fimbriae and S-layer protein [7]. Several studies have indicated that the expression of virulence factors and biofilm formation in *A. hydrophila* are related to bacterial quorum sensing. *A. hydrophila* produces N-acyl-L-homoserine lactones such as N-butanoyl homoserine lactone (BHL) and N-hexanoyl homoserine lactone (HHL) as signal molecules [8]. Disruption of QS in *A. hydrophila* leads to decreased expression of virulence factors [9, 10].

*Bacillus* spp. live in the environment in the air, soil and water, and some of them have been used as probiotics [11]. Previous studies have shown that *Bacillus* sp. strain QSI-1 has probiotic properties and can decrease the pathogenicity of *A. hydrophila* YJ-1 in zebrafish (*Danio rerio*) and Goldfish (*Carassius auratus*) models [12–14]. We hypothesized that QSI-1 plays important roles in altering the physiological characteristics of *A. hydrophila* YJ-1, such as metabolism and virulence factor production. In order to improve the understanding of *Bacillus* sp. QSI-1 effects on *A. hydrophila* physiology, 2-D gel-based and MALDI-TOF/TOF MS-based proteomic techniques were used to compare global protein expression patterns and extracellular virulence factors of *A. hydrophila* samples with and without exposure to QSI-1. This study provides new perspectives on bacteria-bacteria interactions. The results of this study likewise suggest that *Bacillus* sp. QSI-1 can disrupt the virulence of *A. hydrophila* YJ-1.

## Results

### Proteome analysis of whole-cell proteins of *A. hydrophila* YJ-1 co-cultured with *Bacillus* sp. strain QSI-1

**Fig. 1:**
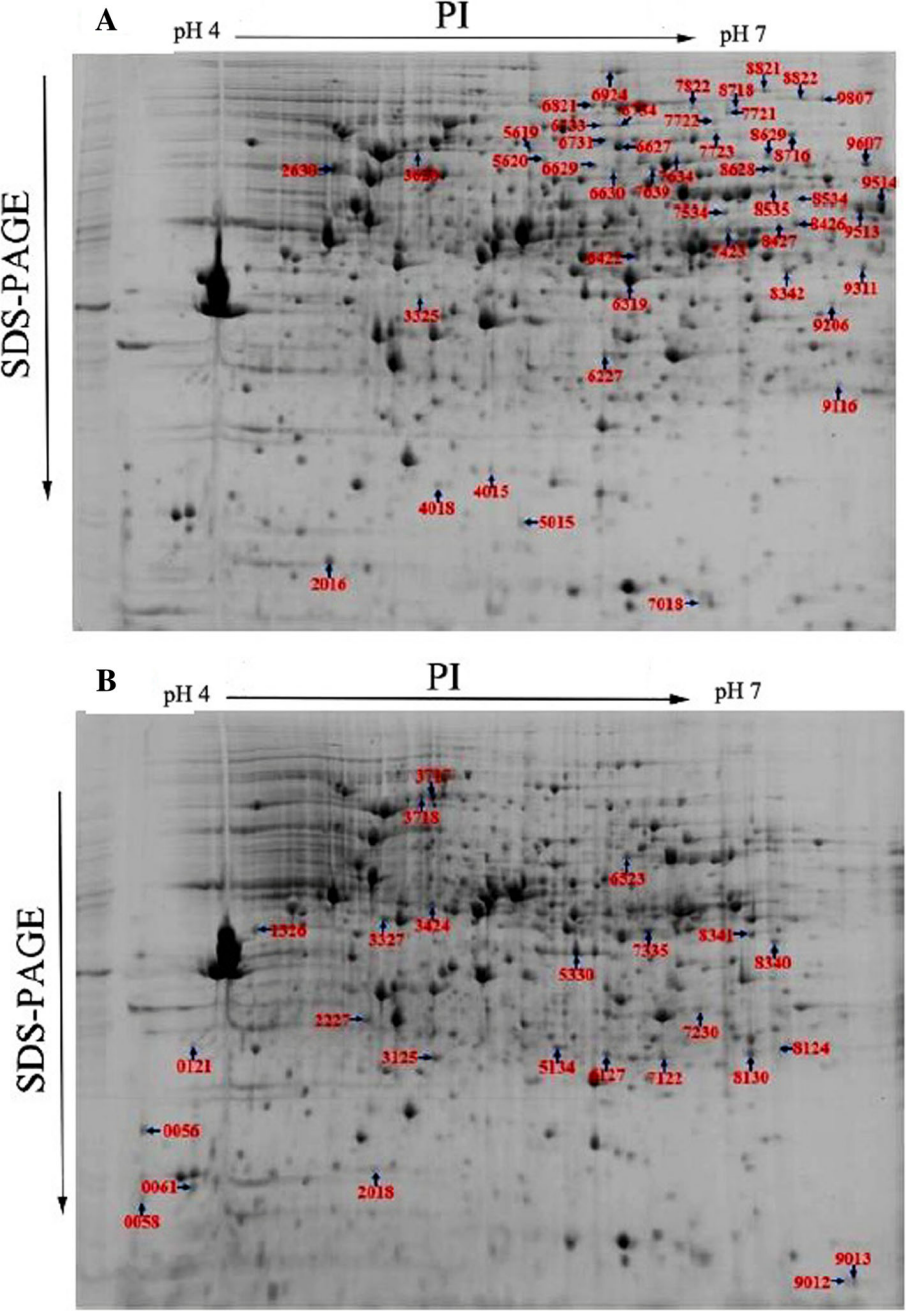
Comparative 2-DE maps of proteins extracted from whole cells of *A. hydrophila*. **a**: mono-culture, **b**: co-culture with QSI-1. Proteins were separated by 2-D electrophoresis using pH 4–7 nonlinear IPG strips and 12% SDS-PAGE. The numbers with arrows indicate the identified protein, and the differentially expressed protein IDs are provided in Table S2

**Fig. 2:**
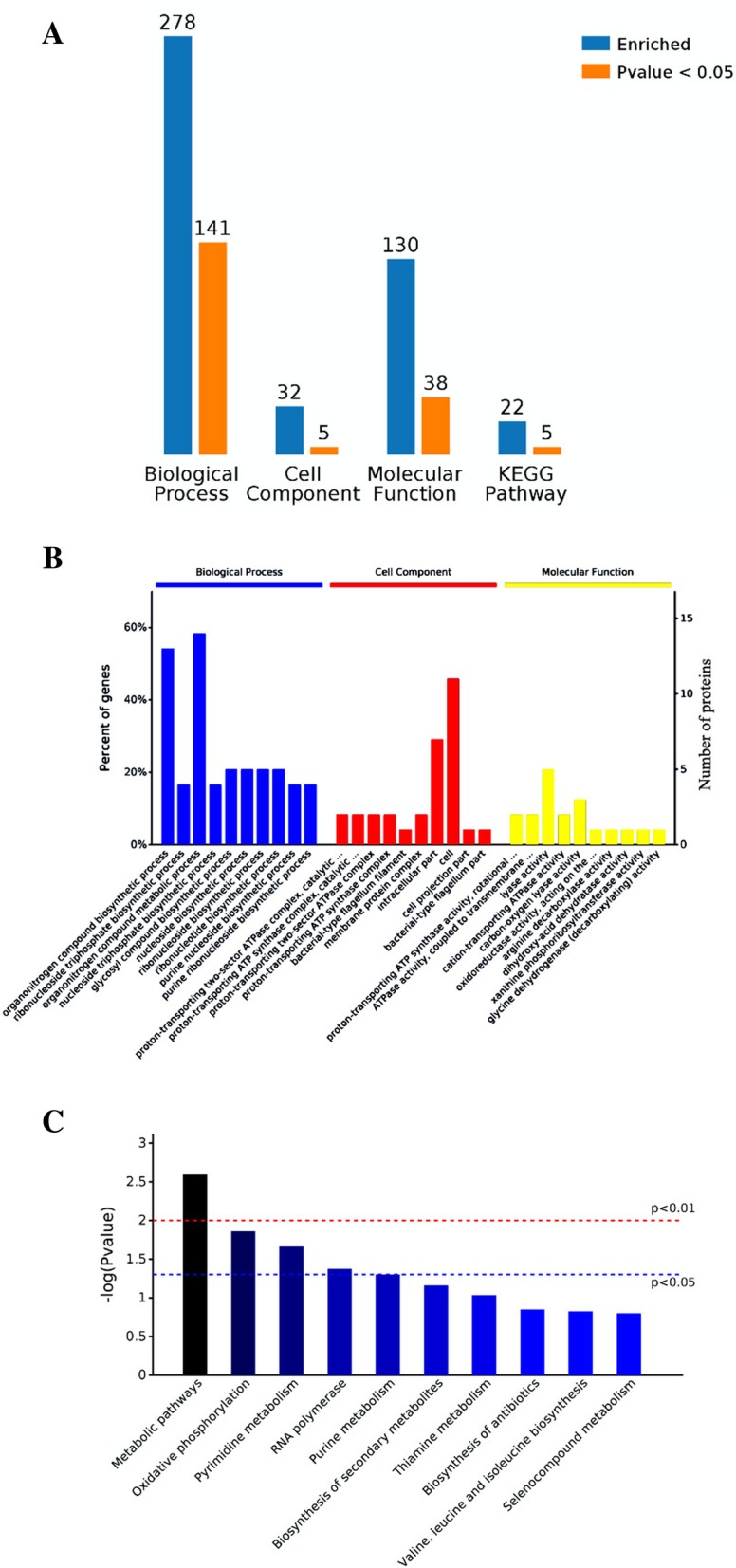
Bioinformatic analysis of the identified differentially expressed proteins. **a** Counts for each category represent the total associated terms in the database with the query protein list. Terms with *P*-values < 0.05 were statistically significant. **b** The ten most significantly enriched terms in level 4 gene ontology hierarchy, information of percentage and number of involved proteins in a term are shown in left and right y-axis. **c** Enriched KEGG pathways are clustered into the metabolism sub-categories; the number of involved proteins in a specific pathway and the corresponding. P-values are shown on the right side of the column

Treatment with probiotics can adjust the gene expression of pathogens, either indirectly via the generation of metabolic molecules or directly via microbe-microbe interactions [15]. To investigate the changes in the *A. hydrophila* YJ-1 whole-cell protein profiles that occurred in co-culture, 2-D electrophoresis maps using IEF on 24 cm, pH 4–7, nonlinear IPG gels was utilized and compared to whole-cell protein profiles of mono-cultures. The results indicated more than 900 protein spots in a pH range of 4–7 on the Coomassie G-250-stained gels (Fig. 1). Quantitative analysis of the three replicates indicated that 72 protein spots demonstrated more than 2-fold-change difference (*P* < 0.05) in expression values compared to the mono-culture. The locations of the over-expressed protein spots were marked with numbers. All 72 spots identified in the 2-D electrophoresis gels were excised, digested and analyzed by MALDI-TOF/TOF MS. After this step, 2 protein spots were not identified (spots 6319 and 8534) and pairs of spots correspond to one ID (14 spots represent 7 protein IDs: spots 0058 and 5015; 3717 and 3718; 8341 and 8342; 5619 and 6734; 8628 and 6627; 8718 and 7721; and 9116 and 8124). Of the 63 identified proteins, only 50 could be classified according to KEGG pathways. The changes in protein expression patterns are shown in the supplementary data (Additional file 1: Table S1). Some of the identified proteins were sub-divided into 22 categories based on comparisons with the KEGG database. A number of metabolic pathways and processes were observed to be similar with the identified proteins. These pathways included metabolic pathways, oxidative phosphorylation pathways, pyrimidine metabolism pathways, RNA polymerase-related pathways, purine metabolism pathways, biosynthetic pathways of secondary metabolites, two-component systems, carbohydrate metabolism pathways, and amino acid and nitrogen metabolism pathways. Among these differentially expressed proteins, 24 and 39 were observed to be increased or decreased, respectively, in *A. hydrophila* YJ-1 co-cultured with QSI-1. To acquire an outline of the elements of differentially communicated proteins that were detected and the potential linkages between them, a Gene ontology (GO) enrichment analysis was performed. In general, 278 proteins were enriched in the biological process (BP) category, with 141 proteins differentially expressed; 32 proteins were marked as cell components (CC), including 5 differentially expressed proteins; a total of 38 differentially expressed proteins were annotated with the molecular function (Fig. 2a). Moreover, the top ten in significantly enriched terms by gene ontology hierarchy (in level 4) were depicted in Fig. 2b. KEGG analysis revealed that most metabolism pathways, including oxidative phosphorylation, pyrimidine metabolism, RNA polymerase activity, and purine metabolism, were significantly improved (Fig. 2c).

### Bacterial growth and extracellular virulence factors of a. hydrophila YJ-1 in mono-culture and in co-culture with QSI-1

**Fig. 3:**
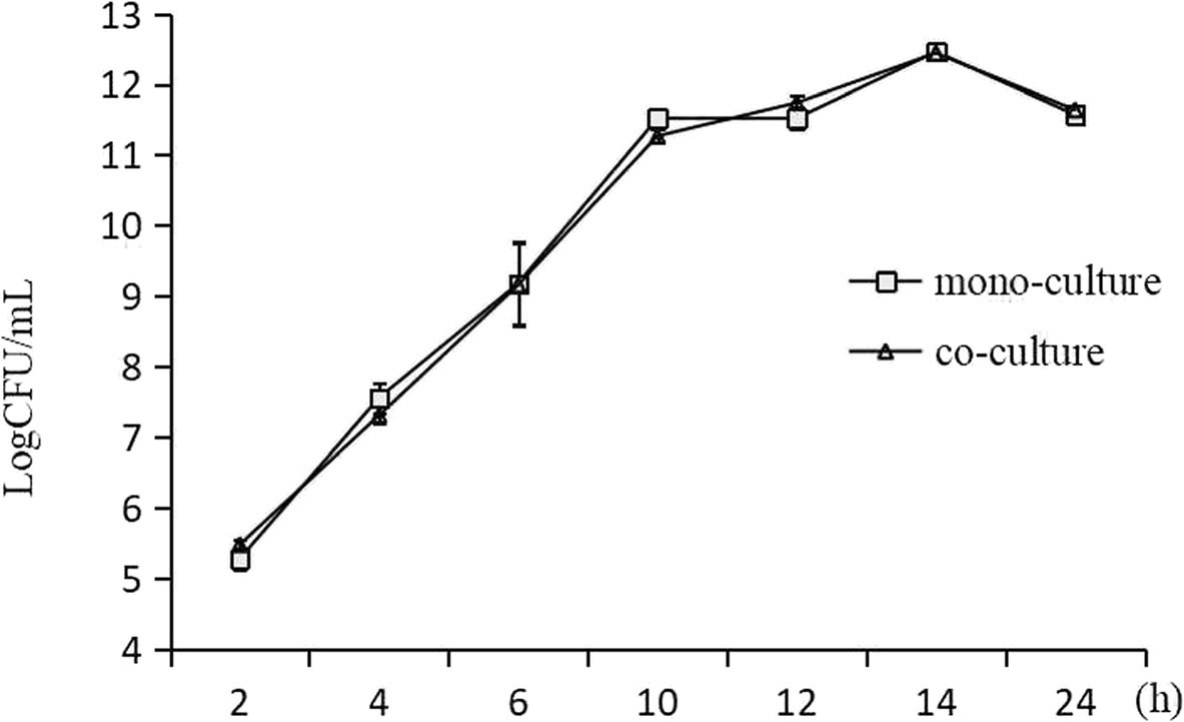
Growth curves of *A. hydrophila* YJ-1 in mono-culture and when co-cultured with QSI-1. The growth rate was measured by the plate counting method. The colony forming units (cfu) were detected from three parallel experiments and are presented as the mean ± standard deviation

**Fig. 4:**
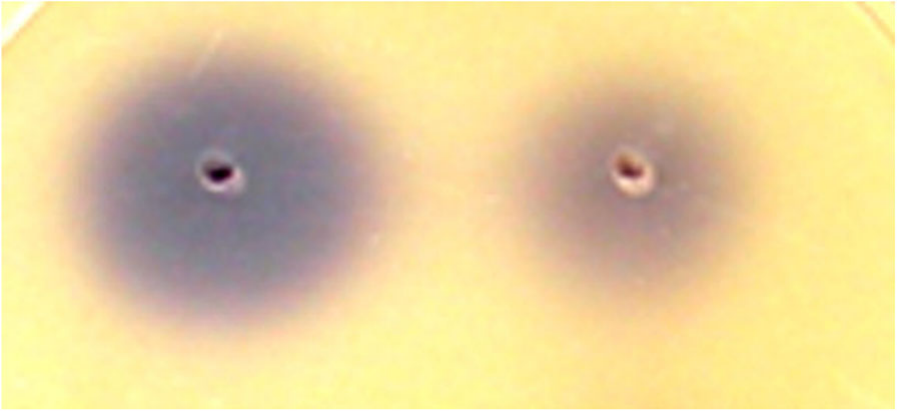
Detection of AHLs activity of culture supernatants. Tests shown on the left are from mono-cultures of *A. hydrophila*, and those on the right are from co-cultures of *A. hydrophila* and *Bacillus* sp. QSI-1

The in vitro growth of the *A. hydrophila* YJ-1 was assessed in mono-culture and co-cultured with QSI-1 using the plate*-*count method. Despite the difference in the inoculum, there were no differences in the CFUs from each bacterium after incubation for 24 h at 28 °C (Fig. 3). Disruption of QS in *A. hydrophila* leads to decreased expression of virulence factors and biofilm formation. Our previous studies have shown that the supernatant of *Bacillus* sp. strain QSI-1 can inhibit the biofilm formation and virulence factors production [10]. In the present study, we used *Transwell* plates to investigate the influence of QSI-1 on the production of virulence factors. We evaluated the effect of *Bacillus* sp. QSI-1 on the production of *A. hydrophila* YJ-1 QS-controlled virulence. As shown in Table 1, the production of hemolysin and protease was drastically inhibited after co-culturing with QSI-1, whereas the lipase activity increased. The AHLs produced by *A. hydrophila* YJ-1 when cultured with or without *Bacillus* sp. QSI-1 was assessed by the production of violacein, as determined by the formation of a zone of purple pigment by *Chromobacterium violaceum* CV026 (Fig. 4). The results indicated that the QS-associated virulence factors and AHLs produced by *A. hydrophila* YJ-1 were significantly altered.

**Table 1:** The extracellular QS-associated virulence factors activity of *A. hydrophila* YJ-1 cultured with *Bacillus* sp. QSI-1

Groups	Diameter of the zone (mm)		
	Hemolysin	Lipase	Protease
*A. hydrophila*	12.83 ± 0.64	10.17 ± 0.40	17.53 ± 0.87
*A. hydrophila + Bacillus* sp.	7.63 ± 0.90	16.70 ± 0.56	9.83 ± 0.71
*P*-value	0.00124	0.00008	0.00029

The diameter of the zone (mm) as a result of QS-associated virulence factors of the supernatant from *A. hydrophila* YJ-1 in mono- and co-culture with *B. subtilis* QSI-1 at 24 h after incubation, respectively, is shown. The experiments were conducted at least three times, and the data are expressed as mean values and standard deviation

### Validation of selected altered proteins via qPCR

**Fig. 5:**
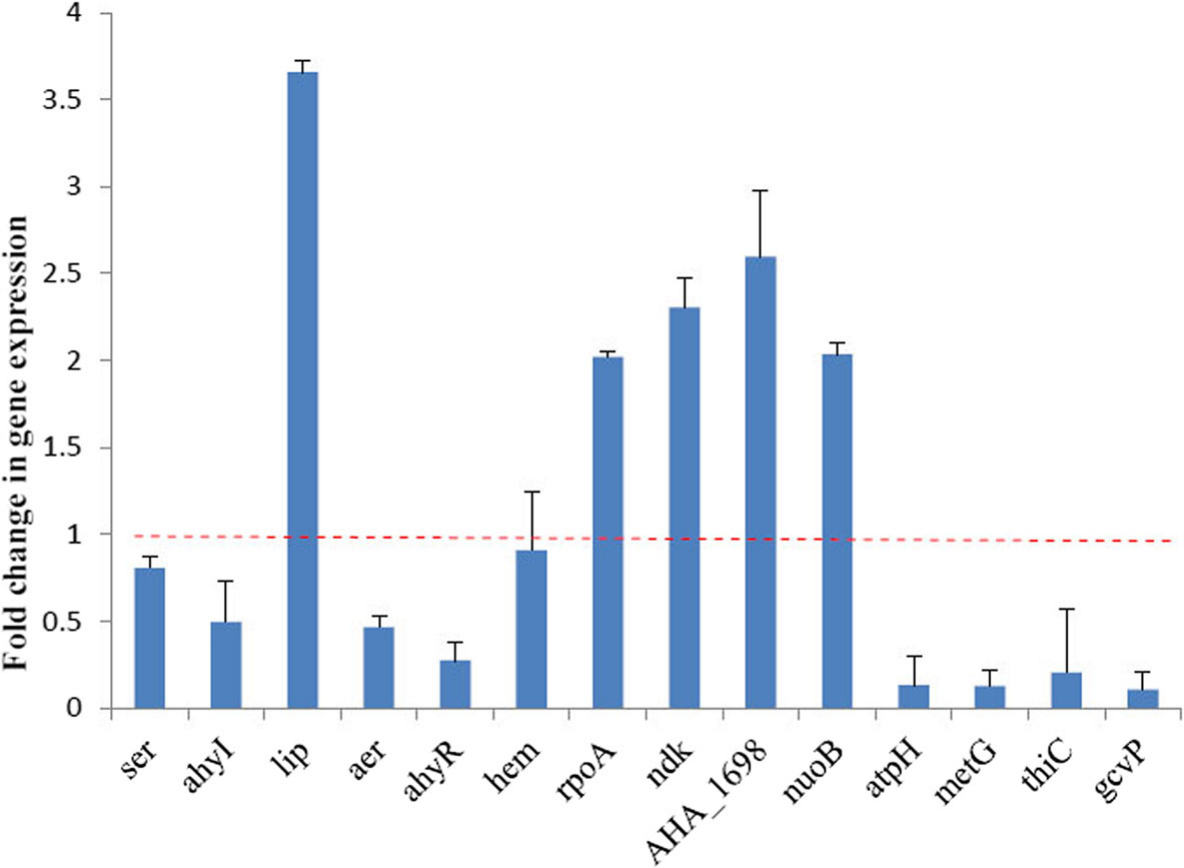
Relative mRNA expression level of selected genes for *A. hydrophila* YJ-1 co-cultured with *Bacillus* sp. QSI-1. The averages of the relative fold change of *A. hydrophila* YJ-1 co-cultured with *Bacillus* sp. QSI-1 compared to that of *A. hydrophila* YJ-1 alone are presented. The error bars represent the standard deviation from three different RNA preparations

We selected a subset of the identified differentially expressed proteins for further validation via qPCR. The qPCR results for the selected proteins were consistent with the proteomic results. The mRNA expression level of quorum-regulated virulence genes *ser*, *hem*, *aer* and *ahyI*, *ahyR* were all decreased; whereas *lip* was transcribed at a significantly higher level in the QSI-1 co-culture compared to that in the mono-culture (Fig. 5). The results suggested that the transcriptional levels of all selected genes significantly changed and was consistent with the results of protein levels.

## Discussion

In natural environments, bacteria rarely live alone and are almost exclusively found in communities with other bacteria. When two different bacterial species are placed together in the same environment, there are at least three possible outcomes from this close encounter: neutralism, competition, and antagonism. QS plays an important role in bacterial interactions. QS systems trigger various responses in bacteria, including motility, biofilm formation, extracellular protease production, secondary metabolism and virulence factor gene expression. Therefore, the inhibition of QS by the degradation of AHL molecules has been proposed as a promising alternative to combat bacterial infections. *Bacillus* sp. QSI-1 can produce quorum-quenching enzymes that can degrade AHLs produced by *A. hydrophila*. The presence of *Bacillus* sp. QSI-1 led to a proteomic response in *A. hydrophila* including the levels of AHLs and QS-regulated extracellular virulence factors. This might be due to the fact that *A. hydrophila* YJ-1 are exposed to quorum quenching enzymes secreted by QSI-1 in the trans-well system. Our results indicated that the QS-associated virulence factors (hemolysin and protease) and AHLs produced by *A. hydrophila* YJ-1 were significantly decreased, only lipase activity increased after co-cultured with QSI-1. The high lipase activity under co-culture conditions may be due to the higher importance of lipase for nutritive resources in competition with others [16]. These results suggested that QSI-1 can influence the phenotypes of *A. hydrophila* because of its production of quorum quenching enzyme. Previous studies have shown that QS plays an important role in the interactions between several species of co-cultured bacteria. The AHL lactonase AiiAAI96 from *Bacillus* can change the metabolic processes within *A. veronii* LP-11 cells and inhibit protease production and motility in *A. veronii* LP-11 [17]. Torabi Delshad et al. [18] showed that co-culture with QQ strains didn’t changed the growth pattern of *Yersinia ruckeri* as measured by CFU, but the swimming motility, biofilm formation, and the production of virulence factors in *Y. ruckeri* were decreased. Our results are reversed with those of the lactic acid bacteria, such as *Bifidobacterium* sp., *Enterococcus mundtii* and *Lactobacillus* sp., which are often used for probiotic applications and the inhibition of the growth of pathogens by producing bactericidal factors [19–21]. A study by Di Cagno et al. showed that variations in the expression of 58 proteins in *Lactobacillus sanfranciscensis* DPPMA174, when co-cultured with *Lactobacillus plantarum* DC400, were affected by interactions with other sourdough *Lactobacilli* through *LuxS*-mediated QS mechanisms [22]. Additionally, a study by Canovas et al. (2016) showed an effect of QS cross-talk on the metabolism and virulence of *Staphylococcus aureus* when co-cultured with *Staphylococcus schleiferi* in the *Galleria mellonella* infection model [23]. A study by Rios-Covián et al. provided new insights into the physiological and molecular mechanisms that determine the interactions of *Bifidobacterium fragilis* and *Bifidobacterium longum* when grown together [24].

This study provides the first insights into the proteomic response of *A. hydrophila* when co-cultured with a quorum-quenching bacterium and uncovers possible molecular mechanisms of probiotic bacteria-pathogen interactions. Results showed that most of the proteins are included in more than one category [25]. Proteins involved in different KEGG pathways affected by other species in co-culture system were also found by other studies. In *Helicobacter pylori* and *Streptococcus mitis* co-culture system, proteins involved in RNA degradation, nucleotide excision repair, mismatch repair, and lipopolysaccharide biosynthesis were increased in co-cultured *H. pylori* [6]. While in *Bifidobacterium longum* and *Bifidobacterium breve* co-culture system, proteins involved in carbohydrate metabolism, gene regulation, cell envelope biogenesis and transport processes were drastically changed [26].

## Conclusion

Our data showed that QSI-1 has a major impact on the expression of proteins, including QS-controlled virulence factors, of *A. hydrophila*. Our results contribute to the knowledge of competition strategies used by *Bacillus* sp. during its co-existence with *A. hydrophila* in natural environment and established new perspectives for the use of *Bacillus* sp. to control *A. hydrophila* infection in aquaculture.

## Methods

### Bacterial strains

The bacterial strains used in this study were *A. hydrophila* YJ-1 [27], *Bacillus* sp. strain QSI-1 [10]. *A. hydrophila* YJ-1 and *Bacillus* sp. strain QSI-1 were cultured in Luria-Bertani (LB) broth medium (Oxoid, United Kingdom) at 37 °C. For long-term storage, the bacterial strains were preserved at − 70 °C in LB containing 15% (v v^− 1^) glycerol.

### Co-culture experiment

Six-well Costar *Transwell* plates (*Costar*, *Corning Inc*., *Kennebunk*, ME, USA) with 0.4 μm polyethylene terephthalate (PET) membrane inserts were used to establish a co-culture system that physically separated the two bacterial species but allowed culture medium with secreted bacterial compounds to be exchanged, as previously described by Khosravi et al. [28], with minor modifications. The co-culture assay of *A. hydrophila* YJ-1 and *Bacillus* sp. QSI-1 cells that grew for 24 h on LB plates were briefly resuspended in LB to OD_600_ values of 0.02 (10^6^–10^7^ cfu ml^− 1^) and 0.008 (10^5^–10^6^ cfu ml^− 1^), respectively. Next, 0.5 mL aliquots of the *Bacillus* sp. QSI-1 and *A. hydrophila* YJ-1 suspensions were added to each well and to the insert well of the 6-well plates, respectively, *six-well Costar plates* were then used for mono-culture. The cultures were incubated at 28 °C for 24 h, and independent experiments were carried out in triplicate.

### Whole-cell protein preparation

Bacterial cells were harvested by centrifugation for 15 min at 4 °C and 12,000 g and then extracted for the whole cell proteins. Whole bacterial cell proteins were prepared as described by Hu et al. and Du et al. [29, 30]. The protein concentration was obtained utilizing the Bradford protein assay (BioRad, CA, USA) with bovine serum albumin (BSA) as a standard according to the manufacturer’s instructions. The samples were stored at − 20 °C until subsequent use.

### Two-dimensional gel electrophoresis, in-gel protein digestion, protein identification and database searches

Two-dimensional gel electrophoresis were performed by the method of Sui et al. [31], IEF was performed using the IPG-phor IEF system (BioRad, CA, USA) and Immobiline 24-cm DryStrip IPG strips (pH 4–7). The 2-D electrophoresis was then performed on a 12% SDS-PAGE. Gel electrophoresis was conducted at 16 °C with 1.0 W gel-1 for 1 h and was later alternated with 10 W gel-1 until the dye formed was approximately 1 cm above the bottom of the gel. The protein signals were visualized by staining the gel with Coomassie Brilliant Blue (CBB) G-250. The gel was imaged with a BioRad Fluor-S system and then analyzed using PDQuest (Version 7.2.0; BioRad) software. Protein spots were first identified and later on coordinated automatically in view of the total densities of the gels. For each of the identified spots, the mean relative volume was thought to be equivalent to its expression level at each stage. Identified spots demonstrating more than 2-fold-change difference (*P* < 0.05) were changed according to the mean relative volume and treated as differentially expressed protein spots. Protein identification by in-gel digestion, mass spectrometric analysis, and database searching using the methods as described in Sui et al. [31].

For GO enrichment analysis UniProt IDs of identified proteins were retrieved from UniProt knowledgebase (UniProtKB) (http://www.uniprot.org/). OmicsBean (http://www.omicsbean.cn), which integrated Gene Ontology (GO) enrichment, Kyoto Encyclopedia of Genes and Genomes (KEGG) pathway analysis, was employed to analyze the obtained differential abundance proteins (http://www.genome.ad.jp/kegg/).

### Detection of extracellular QS-associated virulence factors and enzymatic activities

Twenty-four hours after incubation, the *A. hydrophila* culture (1 mL) was removed from the well of mono- and co-culture plate, and centrifuged at 6000 g for 5 min. The supernatant was filtered through a 0.45-μm pore-size membrane and stored at − 20 °C until further use.

N-acyl-homoserine lactone (AHL) production was detected by the biosensor *Chromobacterium violaceum* CV026. Spent supernatants (10 mL) from stationary phase cultures of *A. hydrophila* YJ-1, from both mono- and co-cultures, were extracted three times with ethyl acetate. The extracts were concentrated to 1 mL, and these concentrated extracts served as exogenous autoinducers and were added to wells inoculated with *C. violaceum* CV026 to test for violacein production. A result was considered positive for the presence of AHLs when the production of violacein, a purple pigment, by *C. violaceum* CV026 was observed, and the diameter of purple pigment was detected.

Extracellular enzyme activities were detected by aliquoting 50 μL of supernatant into wells that were cut into LB agar plates supplemented with different substrates, which were then incubated at 37 °C for 24 h. Protease activity was determined by the production of a clear zone of proteolysis around the wells on 1% skim-milk agar plates [32]. The Hemolysin activity was determined by the production of a zone of hemolysis around the holes on blood agar plates containing 2% (v v^− 1^) sheep blood [33]. The Lipase activity was assayed on Spirit Blue Agar (SBA) (Hi-Media, Mumbai, India) plates, with the presence of lipase visualized as clear blue-colored halos around the wells [26].

### Validation of selected altered proteins at the level of mRNA transcription by quantitative real-time PCR

The cultures were grown in triplicate at 28 °C for 24 h under the conditions previously described. Total RNA was extracted using the RNeasy Mini kit (Qiagen Inc., Valencia, CA), the purity and the concentration of RNA was measured with a NanoDrop 2000 spectrophotometer (Thermo Scientific, UK). We examined the expression levels of 8 significantly changed proteins from proteomic results, and virulence factors such as *Ser* (serine protease), *aer* (aerolysin), *Hem* (hemolysin), *lip* (lipase), and *AhyI/R* (AHLs synthase and receptor genes). The primers used in this study are shown in Additional file 2: Table S2. Relative gene expression was measured by real-time PCR using the ABI 7500 Fast quantitative PCR system (Applied Biosystems, Carlsbad, USA) and FastStart Universal SYBR Green Master Mix (Rox) (Roche, Mannheim, Germany). 16S rRNA gene was used as a reference gene (forward: 5′-GGGAGTGCCTTCGGGAATCAGA-3′, and reverse: 5′- TCACCGCAACATTCTGATTTG -3′) [15]. Amplifications were performed as described by Feng et al. [34]. Deionized water was used as negative controls. Relative quantification was performed using the 2^-ΔΔCt^ method, where ΔΔCt = (Ct target − Ct 16S rRNA) co-culture − (Ct target − Ct 16S rRNA) mono-culture [35].

### Statistical analysis

The data were statistically analyzed using an SPSS software package for Windows version 19.0 (SPSS Inc., Chicago, IL, USA). Differences in the mean values of parameters were tested by oneway analysis of variance and separated by Tukey’s honestly significant difference test (*P* < 0.05).

## Additional files

Additional file 1: **Table S1.** Functional classes and unclassified proteins of *A. hydrophila* YJ-1 that were differentially expressed when co-cultured with *Bacillus* sp. QSI-1. (DOCX 31 kb)

Additional file 2: **Table S2.** Primer sequences for qPCR in this study. (DOC 46 kb)

## Data Availability

All data generated or analyzed during this study are included in this published article and its supplementary information files.

## Abbreviations

2-DE: 2-dimensional gel electrophoresis
*A. hydrophil*: *Aeromonas hydrophila*
AHLs: Acylated homoserine lactones
BHL: N-butanoyl homoserine lactone
BP: Biological process
CBB: Coomassie Brilliant Blue
CC: Cell components
CFU: Colony-forming unit
GO: Gene ontology
HHL: N-hexanoyl homoserine lactone
IEF: Isoelectric focusing
KEGG: Kyoto Encyclopedia of Genes and Genomes
LB: Luria-Bertani
MALDI-TOF/TOF MS: matrix-assisted laser desorption ionization-time of flight mass spectrometry
mRNA: Messenger ribonucleic acid
PET: Polyethylene terephthalate
QQ: Quorum quenching
QS: Quorum sensing
RT-qPCR: Reverse transcription quantitative real-time polymerase chain reaction
SD: Standard deviation
SDS-PAGE: Sodium dodecyl sulfate polyacrylamide gel electrophoresis
