# Supplementary material for: Effect of quorum-quenching bacterium Bacillus sp. QSI-1 on protein profiles and extracellular enzymatic activities of Aeromonas hydrophila YJ-1
Source: BMC Microbiol. 2019 Jun 21;19:135. doi: 10.1186/s12866-019-1515-6 (PMC6588933; doi:10.1186/s12866-019-1515-6)
Supplement: Supplementary file 1 — Table S1. Functional classes and unclassified proteins of A. hydrophila YJ-1 that were differentially expressed when co-cultured with Bacillus sp. QSI-1. (DOCX 31 kb) [file 12866_2019_1515_MOESM1_ESM.docx]

**Table S1.** Functional classes and unclassified proteins of *A. hydrophila* YJ-1 that were differentially expressed when co-cultured with QSI-1.

| Spot Number | Protein ID | Gene Name | Protein Description | Fold Change |
| --- | --- | --- | --- | --- |
| ***Metabolic pathways*** | | | | |
| 3327 | A0KF45 | rpoA | DNA-directed RNA polymerase | 2.121 |
| 6127 | A0KJ66 | nuoB | NADH-quinone oxidoreductase | 2.06 |
| 6523 | A0KQY0 | atpA | ATP synthase | 2.03 |
| 9013 | A0KJ40 | ndk | Nucleoside diphosphate kinase | 2.33 |
| 2016 | A0KQY1 | atpH | ATP synthase | 0.132 |
| 3620 | A0KIP8 | speA | Biosynthetic arginine decarboxylase | 0.213 |
| 4018 | A0KNR2 | gpt | Xanthine phosphoribosyltransferase | 0.37 |
| 6630 | A0KQS4 | ilvD | Dihydroxy-acid dehydratase | 0.435 |
| 6733 | A0KFE6 | thiC | Phosphomethylpyrimidine synthase | 0.195 |
| 7722 | A0KP85 | glgB | 1,4-alpha-glucan branching enzyme GlgB | 0.406 |
| 8629 | A0KGH2 | pyrG | CTP synthase | 0.336 |
| 9311 | A0KKS7 | aroC | Chorismate synthase | 0.221 |
| 9807 | A0KJ05 | gcvP | Glycine dehydrogenase (decarboxylating) | 0.107 |
| ***Oxidative phosphorylation*** | | | | |
| 6127 | A0KJ66 | nuoB | NADH-quinone oxidoreductase | 2.06 |
| 6523 | A0KQY0 | atpA | ATP synthase | 2.03 |
| 2016 | A0KQY1 | atpH | ATP synthase | 0.132 |
| ***Pyrimidine metabolism*** | | | | |
| 3327 | A0KF45 | rpoA | DNA-directed RNA polymerase | 2.121 |
| 9013 | A0KJ40 | ndk | Nucleoside diphosphate kinase | 2.33 |
| 8629 | A0KGH2 | pyrG | CTP synthase | 0.336 |
| ***RNA polymerase*** | | | | |
| 3327 | A0KF45 | rpoA | DNA-directed RNA polymerase | 2.121 |
| ***Purine metabolism*** | | | | |
| 3327 | A0KF45 | rpoA | DNA-directed RNA polymerase | 2.121 |
| 9013 | A0KJ40 | ndk | Nucleoside diphosphate kinase | 2.33 |
| 4018 | A0KNR2 | gpt | Xanthine phosphoribosyltransferase | 0.37 |
| ***Biosynthesis of secondary metabolites*** | | | | |
| 9013 | A0KJ40 | ndk | Nucleoside diphosphate kinase | 2.33 |
| 4018 | A0KNR2 | gpt | Xanthine phosphoribosyltransferase | 0.37 |
| 6630 | A0KQS4 | ilvD | Dihydroxy-acid dehydratase | 0.435 |
| 7722 | A0KP85 | glgB | 1,4-alpha-glucan branching enzyme GlgB | 0.406 |
| 9311 | A0KKS7 | aroC | Chorismate synthase | 0.221 |
| 9807 | A0KJ05 | gcvP | Glycine dehydrogenase (decarboxylating) | 0.107 |
| ***Thiamine metabolism*** | | | | |
| 6733 | A0KFE6 | thiC | Phosphomethylpyrimidine synthase | 0.195 |
| ***Biosynthesis of antibiotics*** | | | | |
| 9013 | A0KJ40 | ndk | Nucleoside diphosphate kinase | 2.33 |
| 6630 | A0KQS4 | ilvD | Dihydroxy-acid dehydratase | 0.435 |
| 9311 | A0KKS7 | aroC | Chorismate synthase | 0.221 |
| 9807 | A0KJ05 | gcvP | Glycine dehydrogenase (decarboxylating) | 0.107 |
| ***Valine, leucine and isoleucine biosynthesis*** | | | | |
| 6630 | A0KQS4 | ilvD | Dihydroxy-acid dehydratase | 0.435 |
| ***Selenocompound metabolism*** | | | | |
| 6731 | A0KKF6 | metG | Methionine--tRNA ligase | 0.126 |
| ***Pantothenate and CoA biosynthesis*** | | | | |
| 6630 | A0KQS4 | ilvD | Dihydroxy-acid dehydratase | 0.435 |
| ***Phenylalanine, tyrosine and tryptophan biosynthesis*** | | | | |
| 9311 | A0KKS7 | aroC | Chorismate synthase | 0.221 |
| ***Arginine and proline metabolism*** | | | | |
| 3620 | A0KIP8 | speA | Biosynthetic arginine decarboxylase | 0.213 |
| ***Glyoxylate and dicarboxylate metabolism*** | | | | |
| 9807 | A0KJ05 | gcvP | Glycine dehydrogenase (decarboxylating) | 0.107 |
| ***2-Oxocarboxylic acid metabolism*** | | | | |
| 6630 | A0KQS4 | ilvD | Dihydroxy-acid dehydratase | 0.435 |
| ***Glycine, serine and threonine metabolism*** | | | | |
| 9807 | A0KJ05 | gcvP | Glycine dehydrogenase (decarboxylating) | 0.107 |
| ***Starch and sucrose metabolism*** | | | | |
| 7722 | A0KP85 | glgB | 1,4-alpha-glucan branching enzyme GlgB | 0.406 |
| ***Flagellar assembly*** | | | | |
| 1326 | A0KIY3 | AHA_1698 | Flagellin | 2.683 |
| ***Biosynthesis of amino acids*** | | | | |
| 6630 | A0KQS4 | ilvD | Dihydroxy-acid dehydratase | 0.435 |
| 9311 | A0KKS7 | aroC | Chorismate synthase | 0.221 |
| ***Aminoacyl-tRNA biosynthesis*** | | | | |
| 5620 | A0KPA5 | proS | Proline--tRNA ligase | 0.285 |
| 6731 | A0KKF6 | metG | Methionine--tRNA ligase | 0.126 |
| ***Carbon metabolism*** | | | | |
| 9807 | A0KJ05 | gcvP | Glycine dehydrogenase (decarboxylating) | 0.107 |
| ***Two-component system*** | | | | |
| 1326 | A0KIY3 | AHA_1698 | Flagellin | 2.683 |
| ***Unclassified protein*** | | | | |
| 8341 | A0A0S3BL09 | AS145_15335 | Lactaldehyde reductase | 10.675 |
| 7230 | A0A0S3BNK6 | mdh | Malate dehydrogenase | 5.576 |
| 2227 | A0A0A6YML4 | AS145_17290 | Uncharacterized protein | 4.474 |
| 0058 | A0A081URM3 | clpP | ATP-dependent Clp protease proteolytic subunit | 3.387 |
| 2018 | A0KFY7 | tpx | Probable thiol peroxidase | 3.011 |
| 3717 | A0A0S3BI43 | AS145_09960 | TonB-dependent receptor | 2.871 |
| 5330 | A0A0A5QEY7 | SH16_02770 | Fructose-bisphosphate aldolase class 2 | 2.681 |
| 7122 | A0A0S3BIC5 | fabG | 3-ketoacyl-ACP reductase | 2.561 |
| 0061 | A0KQ36 | AHA_3953 | Outer membrane protein W | 2.240 |
| 3424 | A0A081UTG4 | A9R12_11940 | Isocitrate dehydrogenase [NADP] | 2.214 |
| 8124 | A0A0F6KC74 | sdhB | Succinate dehydrogenase iron-sulfur subunit | 2.132 |
| 7335 | A0A0J1KBQ2 | SH16_01185 | Uncharacterized protein | 2.123 |
| 8130 | A0A081UTF0 | A9R12_11855 | Uridine phosphorylase | 2.104 |
| 3125 | A0A142E8K6 | deoD | Purine nucleoside phosphorylase DeoD-type | 2.086 |
| 0121 | A0KGK5 | AHA_0854 | Outer membrane porin protein | 2.067 |
| 0056 | A0A0A6CFT6 | tpiA | Triosephosphate isomerase | 2.037 |
| 9012 | A0A0S3BKU3 | AS145_14860 | Cold-shock protein | 2.026 |
| 5134 | A0KJQ6 | AHA_1976 | Electron transfer flavoprotein beta-subunit | 2.016 |
| 8340 | A0A0A5NP40 | SH16_00791 | Uncharacterized protein | 2.009 |
| 6227 | A0A0A5LAZ4 | A0A0A5LAZ4 | Uncharacterized protein | 0.463 |
| 6629 | A0KGU6 | AHA_0950 | ABC transporter, ATP-binding protein | 0.456 |
| 5619 | A0KQ96 | fusA | Elongation factor G | 0.439 |
| 7534 | A0A1C2KVF7 | A9R12_07405 | Methylmalonate-semialdehyde dehydrogenase (Acylating) | 0.424 |
| 7639 | A0A0S3BDR7 | argS | Arginine--tRNA ligase | 0.422 |
| 4015 | A0A081UVD5 | A9R12_20340 | Carbonate dehydratase | 0.421 |
| 9206 | A0A0S3BN19 | AS145_18550 | Uncharacterized protein | 0.418 |
| 7634 | A0A0S3BKX5 | glnS | Glutamine--tRNA ligase | 0.397 |
| 6422 | A0A0S3BEH4 | A0A0S3BEH4 | Cobalamin biosynthesis protein CobW | 0.386 |
| 6821 | A0A1B8Z3I2 | A9R12_09840 | Pyruvate dehydrogenase E1 component | 0.356 |
| 8535 | A0A142E709 | AMS64_17140 | Pyruvate kinase | 0.353 |
| 7018 | A0KJG4 | AHA_1884 | Putative pterin-4-alpha-carbinolamine dehydratase | 0.352 |
| 3325 | A0A0A5QBI9 | SH16_02532 | Peptidyl-prolyl cis-trans isomerase | 0.317 |
| 8821 | A0A0S3BJT7 | AS145_12955 | 2-oxoglutarate dehydrogenase subunit E1 | 0.307 |
| 8716 | A0A081UMY1 | A9R12_15170 | Fumarate reductase flavoprotein subunit | 0.305 |
| 7423 | A0A081USF6 | A9R12_17190 | Homogentisate 1,2-dioxygenase | 0.289 |
| 2630 | A0A1C2KVS1 | A9R12_03430 | Ribosomal protein S12 methylthiotransferase accessory factor YcaO | 0.288 |
| 9513 | A0A081UU94 | nqrA | Na(+)-translocating NADH-quinone reductase subunit A | 0.286 |
| 6627 | W0A052 | W0A052 | PrkA family serine protein kinase | 0.286 |
| 7723 | A0A0A5NJB7 | SH16_02847 | Type II secretion system protein D | 0.230 |
| 7822 | A0A0J1JIU1 | gyrB | DNA gyrase subunit B | 0.223 |
| 7721 | A0A081UQA7 | A9R12_03440 | Formate acetyltransferase | 0.218 |
| 6924 | A0A0S3BK66 | purL | Phosphoribosylformylglycinamidine synthase | 0.209 |
| 9607 | A0A0F6K9J6 | A9R12_00680 | ABC transporter ATP-binding protein | 0.165 |
| 8427 | A0KK94 | AHA_2171 | Protein SsnA | 0.159 |
| 8426 | A0A0S3BGD7 | AS145_06695 | 4-aminobutyrate aminotransferase | 0.152 |
| 9514 | A0A0S3BJE7 | prpD | 2-methylcitrate dehydratase | 0.138 |
| 8822 | A0A081USB1 | A9R12_16965 | Aldehyde-alcohol dehydrogenase | 0.120 |
