# Supplementary material for: Effect of quorum-quenching bacterium Bacillus sp. QSI-1 on protein profiles and extracellular enzymatic activities of Aeromonas hydrophila YJ-1
Source: BMC Microbiol. 2019 Jun 21;19:135. doi: 10.1186/s12866-019-1515-6 (PMC6588933; doi:10.1186/s12866-019-1515-6)
Supplement: Supplementary file 2 — Table S2. Primer sequences for qPCR in this study. (DOC 46 kb) [file 12866_2019_1515_MOESM2_ESM.doc]

**Table S2.** Primer sequences for qPCR in this study.

| **Gene name** | **Primer** | **Sequence (5’-3’)** |
| --- | --- | --- |
| rpoA | F | 5’-GCTGGAAGGCAAGGACGAGGTAAC-3’ |
|  | R | 5’-AACACGAGCAGAAGCAGGCACATAA-3’ |
| ndk | F | 5’-AGCAAGAACCTGATCGGTGCCATC-3’ |
|  | R | 5’-TCTCGGCGTCGGAGAAGAAGTAGG-3’ |
| AHA_1698 | F | 5’-CGCAACGCCAACGACGGTATCT-3’ |
|  | R | 5’-GTCTGACCAGCATCAGCACCAACTT-3’ |
| nuoB | F | 5’-GAGGTGCTGCAAGACACTGTCAACT-3’ |
|  | R | 5’-GGTGAAGGCGGTACACATCTCTACG-3’ |
| atpH | F | 5’-CAAGTAGCGGAGAACGAGACCATC-3’ |
|  | R | 5’-CAAGCGACCATTCTCAGCCATCA-3’ |
| metG | F | 5’-GCTCTATCACTTCATCGGCAAGGAT-3’ |
|  | R | 5’-CTTGGACATCTTGGCACCGTTGA-3’ |
| thiC | F | 5’-ATCGGCAACTCGGCGGTTAC-3’ |
|  | R | 5’-CGGCGTGGATGGTGAAGTAGTC-3’ |
| gcvP | F | 5’-CAGCACGGTGGTCAGGTCTATCT-3’ |
|  | R | 5’-GTGAGGAATGGCGAAGGTCTTGTG-3’ |
| aer (Aerolysin) | F | 5’-GCTATGATGTCACCCTGCGTTACG-3’ |
|  | R | 5’-GATGGAGAGTTCGGTTTCCCCTAC-3’ |
| hem (Hemolysin) | F | 5’-CAAGGGGTTCGTGCCGAATCTGG-3’ |
|  | R | 5’-ATGGGGCGGATGTTGACCGAGGAG-3’ |
| lip (lipase) | F | 5’-GCCGCTGAATCCCTCCTCCTAC-3’ |
|  | R | 5’-TGCTGCCGACGTTGTTCTTGTAG-3’ |
| ahy I | F | 5’-TCTGGAGCAGGACAGTTTCG-3’ |
|  | R | 5’-ATGATGCAGGTCAGTTCGCT-3’ |
| ahyR | F | 5’-TTTACGGGTGACCTGATTGAG-3’ |
|  | R | 5’-CCTGGATGTCCAACTACATCTT-3’ |
